# The impact of surgical treatment for deep endometriosis: metabolic profile, quality of life and psychological aspects

**DOI:** 10.61622/rbgo/2024rbgo42

**Published:** 2024-06-27

**Authors:** Claruza Braga Holanda Lavor, Francisca Adriele Vieira Neta, Antonio Brazil Viana, Francisco das Chagas Medeiros

**Affiliations:** 1 Universidade Federal do Ceará Fortaleza CE Brazil Universidade Federal do Ceará, Fortaleza, CE, Brazil.

**Keywords:** Endometriosis, Laparoscopy, Pelvic pain, Inflammation, Lipids, Anxiety, Depression, Mental health, Quality of life, Surveys and questionnaires

## Abstract

**Objective:**

To evaluate the effects of surgical treatment of deep endometriosis on the metabolic profile, quality of life and psychological aspects.

**Methods:**

Prospective observational study, carried out with women of reproductive age diagnosed with deep endometriosis, treated in a specialized outpatient clinic, from October/2020 to September/2022, at a University Hospital in Fortaleza - Brazil. Standardized questionnaires were applied to collect data on quality of life and mental health, in addition to laboratory tests to evaluate dyslipidemia and dysglycemia, at two moments, preoperatively and six months after surgery. The results were presented using tables, averages and percentages.

**Results:**

Thirty women with an average age of 38.5 years were evaluated. Seven quality of life domains showed improved scores: pain, control and impotence, well-being, social support, self-image, work life and sexual relations after surgery (ES ≥ 0.80). There was an improvement in mental health status with a significant reduction in anxiety and depression postoperatively. With the metabolic profile, all average levels were lower after surgery: total cholesterol 8.2% lower, LDL 12.8% lower, triglycerides 10.9% lower, and fasting blood glucose 7.3% lower (p < 0.001).

**Conclusion:**

Surgical treatment of deep endometriosis improved the quality of life and psychological aspects of patients. The lipid profile of patients after laparoscopy was favorable when compared to the preoperative lipid profile.

## Introduction

Endometriosis is a chronic gynecological and neuroinflammatory disorder affecting about 10% of reproductive-aged women. It is defined by the presence of endometrium tissue outside the endometrial cavity, mainly in the pelvic peritoneum and ovaries.^(1)^ Traditionally characterized by pelvic pain and infertility, it is considered a condition that can significantly compromise women’s quality of life, social relationships, sexuality, and mental health.^(2)^Furthermore, studies show its association with inflammatory response, increased oxidative stress, and atherogenic lipid profile.^(3,4)^

Peritoneal fluid (PF) in endometriosis is characterized by the presence of macrophages with reduced phagocytic capacity, increased pro-inflammatory activation of cytokines, pro-angiogenic factors, and growth factors.^(5,6)^Given this scenario, we can mention two important conditions, atherosclerosis and endometriosis, apparently unconnected, but which put inflammatory markers and lipoproteins together in the peritoneal fluid, with the consequent oxidation of LDL-cholesterol (Low-Density Lipoprotein).^(7,8)^Lipid peroxidation products profoundly affect the growth and aggregation of endometrial cells in the pelvic cavity.^(9)^

A more atherogenic lipid profile involves increased low-density lipoprotein (LDL) and reduced high-density lipoprotein (HDL). This combination increases the frequency of oxidation of LDL particles, which is one of the factors that causes atherosclerosis. Lipid profiles associated with atherosclerosis have been confirmed in plasma and peritoneal fluid (PF) of women with endometriosis, suggesting exudation of plasma components during peritoneal fluid formation. This corroborates the hypothesis that women with endometriosis also have a higher frequency of dyslipidemia, and endometriotic lesions may have similar etiopathogenic substrates triggered by inflammatory processes and oxidative stress.^(10,11)^

A study^(12)^showed that patients with endometriosis reported a higher stress level and higher depression scores, especially those with deep endometriosis. They also observed a significant correlation between age, experience of emotional well-being, control, and social support. Psychiatric disorders can influence gynecological patients’ perception and reporting of symptoms, prognosis, adherence to treatment, and general well-being, influencing the success of therapeutic interventions and quality of life.^(13)^Women with endometriosis may have problems at work, in family relationships, in social life, with self-esteem and mood, with a high rate of psychiatric symptoms, notably depression, anxiety, and increased stress.^(14)^

This research considers the probable associations between endometriosis, changes in metabolic parameters, quality of life, and mental status. Identifying a lifetime link between endometriosis and metabolic disturbances would require a change in public health management. In addition to treatments for infertility and chronic pelvic pain, there would be a need for guidelines for the prevention and early detection of chronic diseases.

## Methods

It is a prospective observational study carried out at the Assis Chateaubriand Maternity Hospital, a reference center for endometriosis treatment in Fortaleza-Brazil, from October/2020 to September/2022. The Research Ethics Committee of the Assis Chateaubriand Maternity Hospital (MEAC) of the Federal University of Ceará (UFC) approved the project, and all patients signed the written informed consent form (WICF).

Women in menacme, over 18 years of age, with a diagnosis of deep endometriosis by ultrasound mapping, with surgical indication, and undergoing the preoperative period were considered eligible to participate in the study. Exclusion criteria: women with grade 2 or 3 obesity (BMI ≥ 35 kg/m^2^); women with a family history of dyslipidemia, diabetes mellitus or systemic arterial hypertension; women using medications such as anxiolytics, antidepressants or mood stabilizers; women who did not have a diagnosis of deep endometriosis confirmed surgically; women who did not respond to the pre- and post-operative questionnaires; women who did not sign the consent form or did not complete all stages of the assessment.

The patients were consecutively selected from the outpatient clinic, and the sample size was determined by the established data collection time. The sample size was calculated using the Gpower 3.1 software, adopting a power of 80% and a significance level of 5%, reaching n = 30. The preoperative diagnosis of endometriosis was given by ultrasonographic mapping performed at the clinic by two professionals with expertise.

Surgical indications followed the service’s protocol: symptoms of pelvic pain unresponsive to clinical treatment in the period between six months and one year; or deep endometriosis with intestinal or ureteral involvement; or presence of ovarian endometrioma > 4 cm. The clinical treatment protocol for endometriosis consisted of using combined oral contraceptives or oral progestogen or levonorgestrel (IUD), but all patients included in the study had already suspended hormonal therapies in the preoperative period for at least three months.

All surgeries performed were by videolaparoscopy (VLP) and included: VLP with exeresis of deep endometriosis foci (with or without intestinal involvement); VLP with exeresis of deep endometriosis foci + oophoroplasty (uni- or bilateral); VLP with exeresis of deep endometriosis foci + hysterectomy; VLP with exeresis of deep endometriosis foci + hysterectomy + oophoroplasty. Hysterectomies were indicated for adenomyosis or uterine leiomyomas occurring with abnormal uterine bleeding and unresponsive to clinical treatment, also observing age and parity factors. Oophoroplasty was indicated for women with endometrioma > 4 cm. The surgeries were performed by two MEAC surgeons with expertise in videolaparoscopy to treat endometriosis.

During the preoperative period, the women initially responded to standardized quality of life instruments (EHP-30) and psychological aspects of anxiety and depression (HADS). The following evaluation was of the metabolic profile and consisted of doing a laboratory test for dyslipidemia (TC; HDL; LDL; Triglycerides (TGC)) and dysglycemia (fasting glycemia). The diagnoses were established with the following parameters.^(15)^

- Dyslipidemia: TC > 200mg/dL, and/or LDL-cholesterol > 130 mg/dL, and/or HDL-cholesterol < 40 mg/dL and/or triglycerides > 150mg/dL;
- Dysglycemia: Fasting glycemia ≥ 100mg/dL;
- Diabetes mellitus (DM): two fasting glycemia measurements ≥ 126 mg/dL or any glycemia ≥ 200 mg/dL.

The EHP-30 was developed by Georgina Jones, validated and translated into Brazilian Portuguese.^(16)^ It is a self-report instrument designed to assess health-related quality of life, specifically for endometriosis, with items developed from interviews with patients and with an established psychometric profile. It consists of a 30-item central questionnaire that applies to all women with endometriosis, referring to five subscales: pain (11 items), control and impotence (6 items), emotions (6 items), social support (4 items) and self-image (3 items); and a 23-item modular questionnaire with six subscales, such as life at work (5 items), relationship with children (2 items), sexual relationship (5 items), relationship with doctors (4 items), treatment (3 items) and infertility (4 items).^(16)^

Response categories are ranked by a five-point Likert Scale (0-4). The raw scores (the sum of the items in each subscale) are translated into an adjusted score (each raw score is divided by the maximum possible raw score and multiplied by 100), ranging from 0 (best possible health status) to 100 (worst possible health status).^(17)^

The Hospital Anxiety and Depression Scale (HADS) was developed by Zigmond and Snaith^(18)^ to identify possible or probable cases of mild anxiety disorders and depression. The scale was translated and validated into Portuguese^(19)^ and contains 14 multiple-choice questions, seven of which are aimed at assessing anxiety (HADS-A) and seven for depression (HADS-D). Each of its items can be scored from 0 to 3, composing a maximum score of 21 points for each scale. The interpretation cut-off point of the HADS scores is nine, and it adopts a theoretical framework so that a score between 0 and 8 means the absence of depressive or anxious symptoms; scores between 9 and 10 mean a possible case of depression or anxiety, and from 11 to 21 as a probable case.^(12)^

To assess the frequency of anxiety and depression, we obtained responses to the HADS items (HADS-A: without anxiety: 0 to 8; with anxiety: ≥ 9; HADS-D without depression: 0 to 8; with depression: ≥ 9). The concept of depression is centered on the notion of anhedonia and is intended to detect mild degrees of mood disorders in non-psychiatric environments to determine the level of anxiety and depression. The questionnaire is short and can be quickly completed by the patient, asking her to respond based on how she felt during the past week.^(20)^

On the two occasions, the Wilcoxon signed-rank test was used to compare the quantitative variables for paired samples. Descriptive statistics of mean and standard deviation expressed the results obtained regarding quantitative variables. A P-value less than 0.05 was statistically significant. The results obtained regarding qualitative variables were expressed through frequencies and percentages. Spearman’s correlation coefficient was calculated to analyze the existence of a correlation between the quality of life and anxiety and depression scores. The McNemar test was applied to observe changes in mental health status categories in both moments. Data were entered into the RedCap Platform, and diagnostic agreement analysis was performed. Statistical analysis was done using the Statistical Package for the Social Sciences (SPSS), version 22.0 (USA), and R 3.3.1 software.

## Results

Of the 72 patients interviewed, 30 met the inclusion and exclusion criteria and completed all assessment steps. All patients had undergone hormone therapy for the clinical treatment of endometriosis prior to surgery for at least six months. Following service protocol, clinical treatment was suspended for 100% of the patients after surgery. Participants’ baseline characteristics are described in table 1.

**Table 1:** Baseline characteristics of study participants

Variables	
Sample size	
Age (age group)	
≤ 40 years	18(60)
41 – 50 years	12(40)
Menarche (years), mean (SD)	11.8 (± 1.5)
BMI (Kg/m^2^), mean (SD)	
Preoperative	31.23 (± 1.91)
Postoperative	30.47 (± 1.50)
BMI (Kg/m^2^)	
Overweight (BMI 25–29.9)	18(60)
Class one obesity (BMI 30–34.9)	12(40)
Blood pressure (mmHg), mean (SD)	
SBP	
preoperative	119 (± 7.8)
postoperative	120 (± 7.1)
DBP	
preoperative	76 (± 5.4)
postoperative	78 (± 5.2)
Infertility	14(46.66)
Parity	
Without children	15(50)
With children	15(50)
HT between 6 months to one year in the preoperative period	
Isolated oral progestogen	14(46.66)
Oral combined estrogen + progesterone	9(30)
Levonorgestrel IUD	7(23.33)
Time to diagnosis (years), mean (SD)	6.2 (± 2.3)
Age at diagnosis (years), mean (SD)	30.1 (±5.2)
Types of surgeries performed	
VLP with exeresis of endometriosis foci	10(33.33)
VLP with exeresis of endometriosis foci + oophoroplasty	7(23.33)
VLP with exeresis of endometriosis foci + hysterectomy	10(33.33)
VLP with exeresis of endometriosis foci + hysterectomy + oophoroplasty	3(10)
Postoperative complications	2(6.66)
Other comorbidities that co-occur with pain	There was no report
Comorbidities (SAH, DM, DLP)	There was no report
Previous diagnosis of Anxiety/Depression and/or medication use	There was no report

mean (± Dp); Caption - BMI (Body Mass Index); SBP - Systolic Blood Pressure; DBP - Diastolic Blood Pressure; IUD - Intrauterine Device; VLP - Videolaparoscopy; SAH - Systemic Arterial Hypertension; DM - Diabetes Mellitus; DLP - Dyslipidemia; HT - Hormonal Therapy

In table 2, we observe that in the comparative evaluation of the general quality of life of women with deep endometriosis in the preoperative period and six months after the surgical procedure, there was an improvement with statistically significant differences in seven domains: pain, control and impotence, emotional well-being, social support, self-image, life at work, and sexual relations. The means of these domains decreased after surgery, thus improving the quality of life score, and we can observe that by measuring the effect size (ES ≥ 0.80: large effect), which measures the magnitude of differences between the quality of life domains at both times.

**Table 2:** Comparison of EHP-30 domains in the preoperative and postoperative periods. Measure of impact between domains and size of effect

Domain score	Preoperative period	Postoperative period.	p^a^	ES
Pain	41.06 ± 15.97 (38.64)	16.43 ± 16.99 (11.36)	< 0.001	0.92
Control and Impotence	56.53 ± 22.47 (58.33)	16.94 ± 19.36 (10.42)	< 0.001	0,97
Emotional well-being	44.31 ± 25.01 (50)	23.33 ± 20.19 (25)	< 0.001	0.82
Social Support	38.75 ± 25.92 (37.5)	24.17 ± 27.16 (18.75)	0.001	0.75
Self-image	46.39 ± 25.3 (50)	27.22 ± 23.77 (25)	< 0.001	0.82
Life at Work	34.67 ± 18.93 (37.5)	12,17 ± 19.81 (0)	< 0.001	0.80
Relation with children	4.17 ± 13.27 (0)	0.83 ± 4.56 (0)	0.265	0.70
Sexual Relations	59.31 ± 19.44 (60)	23.62 ± 21.21 (20)	< 0.001	0.97
Relation with physicians	10.63 ± 14.51 (0)	8.96 ± 15.46 (0)	0.218	0.41
Treatment	19.38 ± 14.8 (18.75)	19.38 ± 14.8 (18.75)	-	-
Infertility	36.88 ± 27.82 (40.63)	33.75 ± 35.07 (25)	0.409	0.28

Data expressed as Mean ± Standard Deviation (Median). a: Wilcoxon test; ES: Effect Size Adjusted EHP-30 score: (raw score ÷ maximum domain score) x 100

In screening for aspects of anxiety and depression using the HADS instrument, using nine as the HADS cut-off point for anxiety and depression, we observed a change regarding anxiety and depression in the sample six months after surgical treatment of deep endometriosis. The means in the preoperative period were 11.13 ± 3.92 and 11.93 ± 4.5, respectively, being classified as a high probability of anxiety and depression. When considering the score six months after surgery, there was a decrease in the mean, 4.83 ± 3.22 and 5.31 ± 3.5, respectively, for anxiety and depression, p < 0.001 (Table 3).

**Table 3:** Comparison of HADS scores for anxiety and depression in the preoperative and postoperative periods

Variables	Preoperative period	Postoperative period	P^a^
HADS anxiety	11.13 ± 3.92 (11)	4.83 ± 3.22 (4)	< 0.001
HADS depression	11.93 ± 4.5 (12)	5.31 ± 3.5 (6)	< 0.001

Data expressed as Mean +/- SD (Median). a) Wilcoxon test Caption: HADS - Hospital Anxiety and Depression Scale

We can also add that, when applying the McNemar test, we observed changes in categories in the preoperative and postoperative periods (6 months after surgery), i.e., patients changed their mental health status. Among 22 patients with depression in the preoperative period, seven patients remained depressed after surgery, and 15 no longer had it after surgery. Likewise, of 22 patients with anxiety in the preoperative period, 19 did not have anxiety after surgery, as described in table 4.

**Table 4:** Change in mental health status observed by the change of HADS categories in the preoperative and postoperative periods in absolute values

Postoperative period			
Preoperative period	With depression (n)	Without depression (n)	p-value*
With depression (n=22)	7	15	< 0.001
Without depression (n=8)	0	8	
	With anxiety (n)	Without anxiety (n)	
With anxiety (n=22)	3	19	< 0.001
Without anxiety (n=8)	1	7	

Data expressed in absolute values, where n is the number of patients; * McNemar test

Regarding laboratory findings, we observed that, in the preoperative period of videolaparoscopy, patients had higher values of total cholesterol, LDL-cholesterol, triglycerides, and fasting glycemia, when compared to six months after surgery, as described in table 5.

**Table 5:** Laboratory variables in the preoperative and postoperative periods

Variables	Preoperative period Mean/sd	Postoperative period Mean/sd	p-value*
TC (mg/dL)	194.5 / 20.08	179.70 / 13.50	< 0.001
LDL (mg/dL)	119 / 20.56	105.60 / 12.18	< 0.001
HDL (mg/dL)	45.50 / 5.04	50.53 / 4.15	< 0.001
TGC (mg/dL)	145.40 /40.79	118.70/19.60	< 0.001
FG (mg/dL)	89.80 / 8.37	83.87 / 5.30	< 0.001

Wilcoxon Test*; Caption - VLP (Videolaparoscopy); CT - total cholesterol; LDL - Low-Density Cholesterol; HDL - High-Density Cholesterol; TGC - Triglycerides; FG - fasting glycemia

## Discussion

Endometriosis is a common chronic gynecological disease, which can lead to a negative impact on the quality of life and mental health of women with this condition. In addition to compromising social and professional relationships, sexuality and reproductive planning, the systemic inflammatory response associated with endometriosis may also predispose these women to a greater spectrum of comorbidities.^(21)^

The main purpose of this study was to evaluate the surgical effect of videolaparoscopy on the improvement of deep endometriosis and its repercussions on quality of life domains, aspects of anxiety and depression, and metabolic profile. When analyzing the scores for the quality of life domains, it was noted that women with deep endometriosis had worse scores, with emphasis on the domains of pain, control and impotence and sexual relations, which is consistent with data from current literature.^(22-27)^

In screening for aspects of anxiety and depression, preoperative averages were high, similar to results by other studies,^(12,28)^who included anxiety and depression as the psychological disorders most associated with endometriosis in their studies. Considering the score 6 months after surgery, we revealed a significant decrease in the averages for anxiety and depression.

Lipoproteins, when compared preoperatively and after surgery, showed a difference between their average levels, with a more substantial difference for LDL and triglyceride levels. Studies^(6,15,29,30)^ also observed an atherogenic lipid profile in women with endometriosis, but future studies are needed to evaluate correlations on the metabolic profile in women with deep endometriosis who underwent laparoscopic surgery and compared with our results.

Other positive aspects reported by participants who showed improvement in pain after surgery were increased willingness to carry out daily activities, increased physical activity, better performance at work and improved sexual intercourse. A study^(26)^ aimed to evaluate pelvic pain and quality of life before and after videolaparoscopy for deep endometriosis, also being significant improvement in the activities mentioned above was observed 1 year after surgery. It was not possible to assess improvement in infertility due to the post-surgery time of 6 months, at which point patients complaining of infertility would begin new attempts to get pregnant.

It is worth noting that the participants in our study had considerably worse quality of life scores preoperatively than after surgical treatment. It is reasonable to assume the pattern of association between endometriosis-related pelvic pain, a frequent symptom, and quality of life. Although we take into account age and delay in diagnosis, social and psychological factors, such as concerns about relationships, fertility and pain related to sexual activity, can also contribute to women’s lower quality of life.

Studies^(20,31)^ observed that psychiatric comorbidity can also influence the assessment of endometriosis, as anxiety, depression and coping methods used can complicate the assessment of chronic pain and diagnosis of endometriosis, with attention to psychological treatment being effective in helping women cope with pelvic pain. Moreover, they highlighted that endometriosis involves an initial rupture, an interruption of normal life, for almost all women in various areas of life, such as work and intimate relationships. However, some women are able to restore a reorganized identity and life meanings, which therefore leads to more positive mental health outcomes. In this process, the emotional support provided by the intimate partner represents an important protective factor.

The endometriosis patients in our study also had an unfavorable lipid profile. Although there was a difference between the mean levels of all lipoproteins when compared preoperatively and after surgical treatment, the difference was more substantial for LDL and triglyceride levels. There was significant improvement after surgical treatment of endometriosis, with a significant decrease in mean levels of TC (8.2% lower), LDL (12.8% lower), TGC (10.9% lower) and FPG (7.3% lower), in addition to an increase in HDL levels (9.9% higher), as shown in the results.

An atherogenic lipid profile in women with endometriosis was detected,^(32)^with TC, LDL and TG levels being higher, and HDL levels lower among patients with endometriosis, an observation also confirmed in the present study. However, these researchers included women with minimal endometriosis in their study, while our study involved patients with moderate or severe endometriosis, and we also did not withdraw hormonal medications before determining the lipid profile, a fact that may represent a bias.

It was also demonstrated^(33)^that endometriosis is involved in changes in the lipid profile and the development of atherosclerotic disease, due to its involvement with oxidative stress and chronic inflammation. This was confirmed by a large study,^(30)^in which women with endometriosis had a higher risk of association with dyslipidemia, high blood pressure and atherosclerosis, and was related to the large amount of circulating inflammatory mediators.

Differently, other authors^(34,35)^ did not detect differences in the lipid profile of women with endometriosis, but their study included patients who were not using hormonal medication (oral hormonal contraceptive, GnRH analogue) for the treatment of endometriosis, which may be an additional confounding factor to be considered in data analysis. Perhaps our study has a limitation in the analysis of the lipid profile, since it analyzed women with endometriosis undergoing hormonal treatment before surgery, and without hormonal treatment after surgery.

Some limitations of our study should be addressed here. The incidence of depression and anxiety disorders may have been overestimated, since the HADS instrument consists of self-assessment of mood, suggesting aspects of these morbidities in non-psychiatric outpatients. We also did not investigate the association between the use of hormonal therapy for the clinical treatment of endometriosis, dyslipidemia and increased risk of depression and anxiety disorders.

The scores obtained in this sample are likely a reflection of the recruitment strategy used in this study, with a sample over-representative of those women presenting with more severe symptoms of endometriosis, and under-representative of those with minimal to mild symptoms. This needs to be taken into account when interpreting the findings. Furthermore, the majority of women indicated experiencing at least some level of anxiety or depression, supporting the findings of previous studies reporting increased levels of anxiety/depression in women with endometriosis.^(28,31)^

We can even report the small sample of patients studied, and an evaluation time of only 6 months after surgery. Among the strengths we had the possibility of working with a specific group of women with deep endometriosis, using validated and specific instruments to assess quality of life and psychological aspects, also combining metabolic assessment of these women.

The aim is to improve care and management for this group of patients, in an attempt to facilitate the path to correct diagnosis and access to effective treatment. Although we did not find a statistically significant association between variables such as age group, relationships, history of infertility, among others, and anxiety and depression, the ideal of the research was of great relevance.

Finally, this study represents an analysis of representative metabolic domains for the female population, observing the relationship between endometriosis and dyslipidemia, and suggesting the early identification of young women at risk for vascular disease and prone to atherogenesis, and with compromised social relationships and professionals, sexuality and reproductive planning.

## Conclusion

Surgical treatment for deep endometriosis improved the metabolic profile, quality of life, anxiety, and depression scores of the women in the study. The lipid profile of the patients six months after surgical treatment for deep endometriosis was favorable when compared to their preoperative lipid profile, with improvement in mean levels of LDL-cholesterol, HDL-cholesterol, total cholesterol, and triglycerides, in addition to improvement in glycemic levels. There was an improvement in quality of life in most of the EHP-30 domains, except for the relationship with children, relationship with physicians, and infertility. There was also a positive change in mental health status, in the pattern of anxiety and depression six months after surgery for deep endometriosis.
